# Evaluative Processing of Food Images: Longer Viewing for Indecisive Preference Formation

**DOI:** 10.3389/fpsyg.2019.00608

**Published:** 2019-03-20

**Authors:** Alexandra Wolf, Kajornvut Ounjai, Muneyoshi Takahashi, Shunsuke Kobayashi, Tetsuya Matsuda, Johan Lauwereyns

**Affiliations:** ^1^Graduate School of Systems Life Sciences, Kyushu University, Fukuoka, Japan; ^2^Brain Science Institute, Tamagawa University, Tokyo, Japan; ^3^Department of Neurology, Fukushima Medical University, Fukushima, Japan; ^4^Faculty of Arts and Science, Kyushu University, Fukuoka, Japan

**Keywords:** gaze duration, viewing time, self-paced versus time-controlled, non-exclusive versus exclusive, evaluative processing, naturalistic food images

## Abstract

The well-known gaze cascade hypothesis proposes that as people look longer at an item, they tend to show an increased preference for it. However, using single food images as stimuli, we recently obtained results that clearly deviated from the general proposal that the gaze both expresses and influences preference formation. Instead, the pattern of data depended on the self-determination of exposure duration as well as the type of evaluation task. In order to disambiguate how the type of evaluation determines the relationship between viewing and liking we conducted the present follow-up study, with a fixed response set size as opposed to the varying set sizes in our previous study. In non-exclusive evaluation tasks, subjects were asked how much they liked individual food images. The recorded response was a number from 1 to 3. In exclusive evaluation tasks, subjects were asked for each individual food image to give one of three response options toward a limited selection: include it, exclude it, or defer the judgment. When subjects were able to determine the exposure duration, both the non-exclusive and exclusive evaluations produced inverted U-shaped trends such that the polar ends of the evaluation (the positive and negative extremes) were associated with relatively short viewing times, whereas the middle category had the longest viewing times. Thus, the data once again provided firm evidence against the notion that longer viewing facilitates preference formation. Moreover, the fact that non-exclusive and exclusive evaluation produced similar inverted U-shaped patterns suggests that the response set size is the critical factor that accounts for the observations here versus in our previous study. When keeping the response set size constant, with an equal opportunity to observe inverted U-shaped patterns, the findings are suggestive of a role for the level of decisiveness in determining the length of viewing time. For items that can be categorically identified as positive or negative, the evaluations are soon completed, with relatively brief viewing times. The prolonged visual inspection for the middle category may reflect doubt or uncertainty during the evaluative processing, possibly with an increased effort of information integration before reaching a conclusion.

## Introduction

A common view with respect to the processes of preference formation holds that looking facilitates positive evaluation. Arguably the best-known phenomenon in this respect is the “gaze cascade effect,” first demonstrated by Shimojo et al. (2003), and replicated numerous times by different labs (including our own) with a range of variations (Glaholt and Reingold, 2009, 2011; Schotter et al., 2010; Morii and Sakagami, 2015; Onuma et al., 2017; Zommara et al., 2018). In the gaze cascade effect, subjects gradually tend to gaze more likely at the item that they will choose, an effect that typically emerges half a second or longer before the actual decision. However, this connection between viewing and liking is obtained using forced-choice tasks with two or more simultaneously presented items, in self-paced viewing conditions. In a recent investigation (Wolf et al., 2018), we questioned to what degree this connection can be generalized to other types of evaluation.

We found that the nature of the task had a major impact on whether and how viewing is connected to preference formation (Wolf et al., 2018). Generally speaking, such research contributes to our understanding of how the type of evaluation may have a critical impact on the information processing during the development of preferences. As such, this research aims to improve our ability to track the mechanisms that underlie evaluative decision-making. The theoretical and practical relevance of such improved tracking resides in being able to tell how the nature of the question may lead the search for an answer. Ultimately, the goal is to protect people’s choices from unwarranted external influences (e.g., due to how the evaluation prompts are framed with different response options).

Reviewing previous research, we noted that the forced-choice task with multiple items in a display sets up a form of *relative* evaluative processing, in which subjects engage in direct comparison among different visual items. The gaze may contribute to fixation-dependent coding of relative value, pulling one item to the foreground at the expense of the others in the display (cf. Lim et al., 2011; Sokol-Hessner et al., 2012). A related problem here is that the forced-choice task with multiple items requires a spatial response, implying that the gaze may act as a spatial precursor to the choice. In other words, the gaze may express spatial preparation rather than preference formation (Van der Laan et al., 2015; Gerbella et al., 2017). To avoid these issues, and to test the generalizability of the connection between viewing and liking, we decided to design an evaluation paradigm on the basis of single images. We opted to use naturalistic food images as stimuli because our research group is currently focused on an extensive project to investigate the development and expression of preferences for food as a critical aspect of health and well-being.

In our previous study (Wolf et al., 2018), we examined the relationship between viewing and liking in four different tasks, using a 2 × 2 design, with two exposure conditions (self-paced versus time-controlled) and two types of evaluation (non-exclusive versus exclusive). The non-exclusive type of evaluation did not impose any restriction on the number of positive evaluations given by subjects. In contrast, in the exclusive type of evaluation, the subjects could give a positive evaluation (inclusion) only to a restricted number of items (a maximum of 15 out of 80 images).

The results clearly deviated from the general proposal that longer viewing leads to more liking. Instead, we found that in time-controlled evaluation tasks, when the subjects were not able to determine the length of time the images were available for viewing, there relationship between viewing time and evaluation turned out to be not significant. Furthermore, even with self-paced viewing, we found the relationship between viewing and liking to be dependent on the type of evaluation. In the non-exclusive evaluation task, we observed an inverse U-shaped relation, with extreme ratings (either very high or very low) associated with shorter viewing times, suggesting a connection between speed and strong opinion. In contrast, in the exclusive evaluation task, longer viewing times were associated with a higher likelihood of inclusion.

Although the pattern of results urgently required a reevaluation of the gaze cascade hypothesis, our previous study (Wolf et al., 2018) also raised several new questions. In the first instance, the pattern of results pointed to a critical difference as a function of the type of evaluation. Accordingly, one interpretation for the diverging results in the non-exclusive versus exclusive evaluation tasks was that putting a cap on the number of positive evaluations effectively changed the nature of the processing. For instance, with a limitation, the evaluation procedure includes opportunity costs (Hayden et al., 2011; Wikenheiser et al., 2013; Constantino and Daw, 2015). Every item included fills a slot in the selection, reducing the number of available slots for the other items. Conversely, there are no such opportunity costs in the non-exclusive evaluation.

The presence of opportunity costs would change the nature of processing by forcing a comparison against a criterion. The exclusive evaluation would then involve a form of cumulative evaluative processing in which the decision is only activated when the internal representation in favor of a decision reaches the critical threshold (e.g., Krajbich et al., 2010). The gaze serves to facilitate this cumulative processing, leading to a connection between viewing and liking, exactly as suggested in the original gaze cascade hypothesis. The only difference when viewing single images would be that this cumulative processing is evaluated against an abstract internal threshold rather than a concrete visual alternative. The exclusive evaluation might still require cumulative processing to activate a decision, implying that viewing leads to liking. This would not be the case in the non-exclusive evaluation, since there is no limitation on the decision-making. In other words, there is no associated risk or opportunity cost when giving a high evaluation to any particular item; every item can be liked without any consequences for other items.

However, in our previous study (Wolf et al., 2018), the diverging results in the non-exclusive and exclusive evaluation tasks could also be due to a completely different factor. The non-exclusive evaluation involved selecting one out of five possible response alternatives (rating from 1 to 5). In contrast, the exclusive evaluation involved selecting one out of two possible response alternatives (include or exclude). It was therefore possible that the response set size had influenced the range of observations. Particularly, an inversed U-shape was from the outset unobservable in the exclusive evaluation simply because there were only two alternatives in that situation. More specifically, it was possible that the relatively long viewing times for inclusion responses in the exclusive evaluation were due to a contamination between fast viewing times for very highly evaluated items and slower viewing times for moderate-to-somewhat-highly evaluated items. Creating a third, moderate response option could then enable the observation of an inverted U-shape also in the exclusive evaluation task.

In order to disambiguate whether the diverging pattern of data was determined by the presence of opportunity costs or by the response set size, we decided to conduct the present follow-up study. Our new study used the same 2 × 2 design, with the same materials and procedures, making only one fundamental change. This time we controlled the response set size so that there were three response options in all tasks. In the non-exclusive evaluation tasks, subjects were asked to rate how much they liked the items from 1 (‘*not at all*’) to 3 (‘*very much*’). In the exclusive evaluation tasks, subjects were instructed to judge for each food image whether they would include it in their selection, exclude it, or defer the judgment.

As in our previous study, we expected that there would be no relationship between viewing and liking in the time-controlled evaluation tasks. With respect to the self-paced evaluation tasks, if opportunity costs critically determine the relationship between viewing and liking, there should be a divergent pattern of results for the exclusive versus non-exclusive evaluation tasks, with viewing leading to liking in the exclusive evaluation task, but not in the non-exclusive evaluation task. In contrast, if the earlier observation of a divergent data pattern was due to the response set size, we should obtain inverted U-shapes in the present study, with longer viewing times for the middle category in both the non-exclusive and exclusive evaluation tasks.

## Materials and Methods

### Subjects

All 40 subjects were students from Kyushu University (12 females and 28 males; with a mean age of 23.70 ± 3.24 years). None of the subjects had participated in our previous study (Wolf et al., 2018). The subjects had normal or corrected-to-normal vision. No subject reported a diagnosis of any eating disorder, sleep deprivation or past or present neuropsychological disorder. The study was conducted in accordance with the ethical principles of Kyushu University. Written informed consent was obtained from each subject. All students received either course credit or a monetary reward of 1,000 yen for their participation.

### Stimuli

**FIGURE 1:**
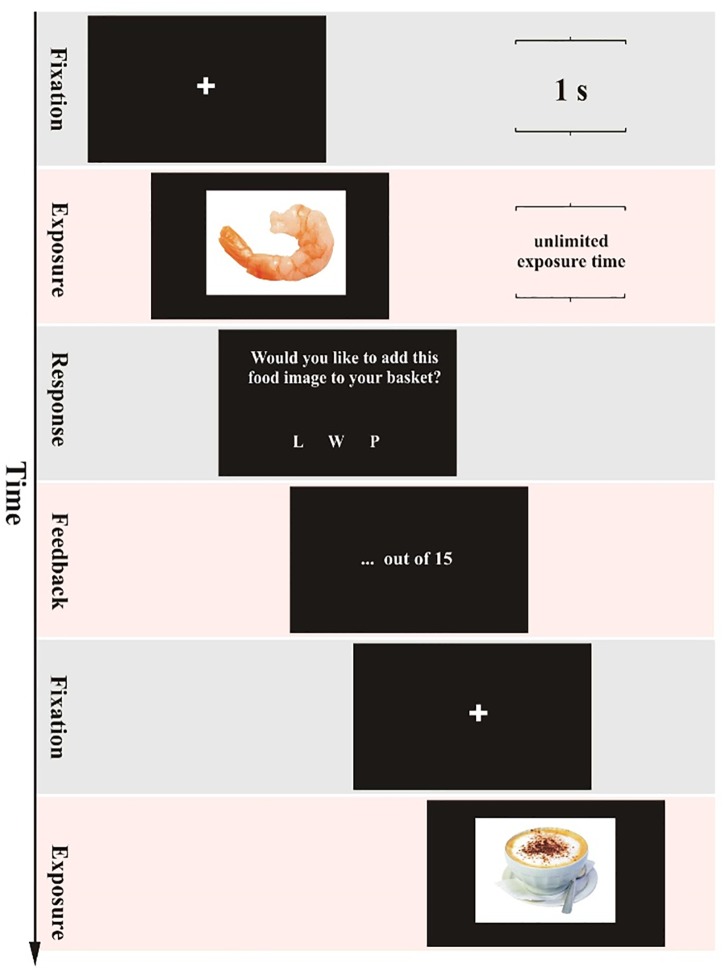
The trial structure in the self-paced exclusive evaluation. The structure was the same in the three other evaluation tasks except for the following critical differences. First, the duration of the exposure frame (second row) was either self-paced or time-controlled. In the self-paced evaluation tasks, the subject had to press the spacebar to proceed to the response frame. In the time-controlled evaluation tasks, the response frame replaced the exposure frame automatically after a computer-generated duration. Second, in the non-exclusive evaluation tasks, the response frame presented the question, “How much do you like this food image?” and gave three response options “1,” “2,” or “3.” The feedback frame indicated the chosen response option.

Stimuli consisted of a set of 320 naturalistic food images with a resolution of 600 × 450 pixels (96 dpi, sRGB color format). The set of images was exactly the same as in our previous study (Wolf et al., 2018), and had been drawn from a food-pictures database for experimental research (Blechert et al., 2014). The set of images was divided into 4 subsets of 80 pictures that showed no significant differences in any of the objective or subjective characteristics of the food-pictures database. For each subject, a different set of 80 pictures was used in each of the four evaluation tasks (see Figure 1 and below for the definition of tasks). Thus, we ensured that subjects were never exposed to the same food image twice. The allocation of picture sets to tasks was counterbalanced; the order of food pictures was randomized within each task; and the order of the tasks was counterbalanced across subjects. Images were presented as a single stimulus on a black background.

### Apparatus

The experimental set-up was exactly the same as in our previous study (Wolf et al., 2018). The visual stimulus was presented on a 23.8-inch FHD flat-panel-monitor, with a display resolution of 1920 × 1080 pixels. The subjects were seated approximately 65 cm from the monitor. To minimize head movement a chin-rest with a forehead-support was used. For all 40 subjects, we recorded manual (keyboard) responses as well as gaze position using Eye Tribe, an eye-tracking device at 60 Hz sampling rate (The Eye Tribe Aps, Denmark); a system with sufficient reliability for present purposes (Ooms et al., 2015; Ounjai et al., 2018; Zommara et al., 2018).

Before the start of a session with eye tracking, the subject was asked to follow a dot on the screen for a 12-point calibration. After the calibration, the gaze coordinates were calculated through Eye Tribe with an average accuracy of less than 0.5° visual angle on a 24-inch display. To prevent heat buildup a small USB fan was used. All events and recordings were controlled through code written in Psychopy (version 1.84.2); for reference, see Peirce (2007, 2009).

To compute actual viewing time (time with eye position on the displayed naturalistic food image) raw data were filtered. First, eye positions beyond the presentation area were removed. Second, detected and recorded eye blinks were also removed from the amount of actual viewing time if they lasted longer than 50 ms. Finally, the obtained data were plotted using custom software, and statistical analyses were conducted.

### Design and Procedure

As in our previous study (Wolf et al., 2018), one experimental session consisted of four different evaluation tasks: two different types of decision (non-exclusive versus exclusive) performed under two different types of exposure duration (self-paced versus time-controlled). Each subject was asked to participate in each of the four evaluation tasks.

Based on the analysis of our previous study (Wolf et al., 2018), we aimed for 40 subjects, with eye-tracking data, as a suitable sample size for present purposes. The subjects were instructed to evaluate the appetitive appeal of the food images; this type of evaluative processing presumably involves a combination of individual food preferences and the esthetic properties of the images. However, in contrast to our previous study, the response set size was kept constant at three response options in all evaluation tasks.

In the non-exclusive evaluation tasks, the subjects were asked to rate how much they liked each food image with three options, either 1 (‘*not at all*’), 2 (neutral), or 3 (‘*very much*’), by pressing the corresponding number on the keyboard. There was no limitation on the number of positive or high evaluations.

In the exclusive evaluation tasks, the subjects were asked to pick a maximum of 15 food images for a virtual “basket,” with three options for every food image, ‘*leave it*’ (i.e., rejection), ‘*wish list*’ (i.e., deferment), and ‘*put it*’ (i.e., inclusion), by pressing the corresponding letter on the keyboard (L for ‘*leave it*,’ W for ‘*wish list*,’ and P for ‘*put it*’). Importantly, this procedure introduced a limitation on the number of positive or high evaluations. Our previous study (Wolf et al., 2018) as well as an earlier pilot study (Espinoza Torres, 2015) had shown that more than 90% of subjects picked the maximum of 15 items and viewed at least 30 items, offering a larger sample of rejected than included items. In the present study, it should be noted that items on the ‘*wish list*’ were considered to be excluded from the virtual basket as soon as the subject had reached the maximum of 15 inclusions. In case the subject did not reach the maximum of 15 inclusions, the items on the ‘*wish list*’ were included in the virtual basket following the trial order (i.e., “first on the wish list, first in the basket”) until the maximum of 15 items was reached.

In the self-paced evaluation tasks, the subjects could determine the length of time they viewed the images, indicating their readiness to move on to their evaluation after viewing the food image by pressing the spacebar. In contrast, in the time-controlled evaluation tasks, the exposure duration was computer-generated, with the food image on the stimulus screen displayed for a pseudo-randomly chosen duration between 1 and 8 s, and then automatically replaced by the response screen.

All other aspects of the procedure were exactly the same as in our previous study (Wolf et al., 2018), and for brevity are not reproduced here.

## Results

### Overall

All subjects (*N* = 40) completed 80 trials in both the self-paced non-exclusive evaluation and the time-controlled non-exclusive evaluation. All subjects completed the self-paced exclusive evaluation (number of items picked for the basket: *M* = 14.28, *SD* = 1.50; number of items put on the wish list: *M* = 13.58, *SD* = 8.35; number of items viewed: *M* = 56.98, *SD* = 18.88). All subjects completed the time-controlled exclusive evaluation (number of items picked for the basket: *M* = 14.53, *SD* = 1.83; number of items put on the wish list: *M* = 12.10, *SD* = 9.20; number of items viewed: *M* = 50.35, *SD* = 16.11). We were able to obtain sufficient-quality eye-tracking data from 39 subjects in the self-paced non-exclusive task; 38 subjects in the time-controlled non-exclusive task; 38 subjects in the self-paced exclusive task; and 37 subjects in the time-controlled exclusive task.

### Self-Paced Non-exclusive Evaluation (SPN)

#### SPN Exposure Time

**FIGURE 2:**
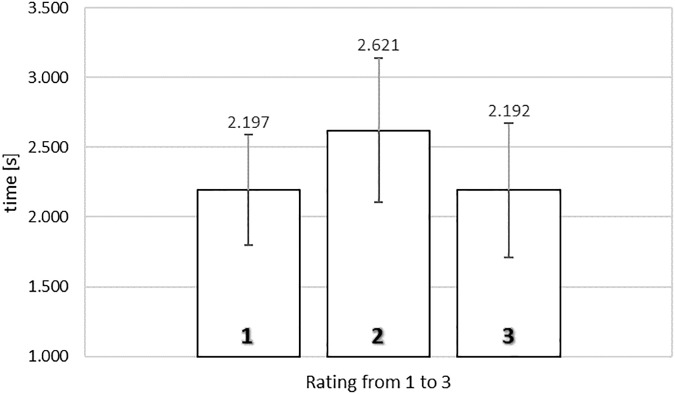
Average exposure time of food images rated from 1 (‘*not like at all*’) to 3 (‘*like very much*’) in the self-paced non-exclusive evaluation. Error bars reflect the 95% confidence interval around the mean.

Figure 2 shows the average exposure times of food images as a function of rating in the self-paced non-exclusive evaluation. To analyze the relationship between rating and exposure time, a one-way repeated measures ANOVA was conducted with three levels of rating (from 1, *‘not like at all*,’ to 3, ‘*like very much’*), using the average exposure times for each subject for each level of rating as dependent measure. Mauchly’s test of sphericity indicated that the assumption of sphericity had not been violated, χ^2^(2) = 2.445, *p* = 0.295.

We observed a significant relationship between rating and exposure time, *F*(2,78) = 8.126, MSE = 0.299, $\eta_{p}^{2}$= 0.172, *p* < 0.005. To gain further insights in the observed relationship between rating and exposure time, we analyzed the polynomial contrasts. The quadratic contrast was significant, *F*(1,39) = 20.598, MSE = 0.236, $\eta_{p}^{2}$= 0.346, *p* < 0.0001, but not the linear contrast, F < 1. The data reflected an inverted U-shape tendency, where images that obtained extreme ratings were associated with shorter exposure durations (2.197 s for a rating of 1; 2.192 s for a rating of 3) than images that received the middle rating (2.621 s).

#### SPN Actual Viewing Time

**FIGURE 3:**
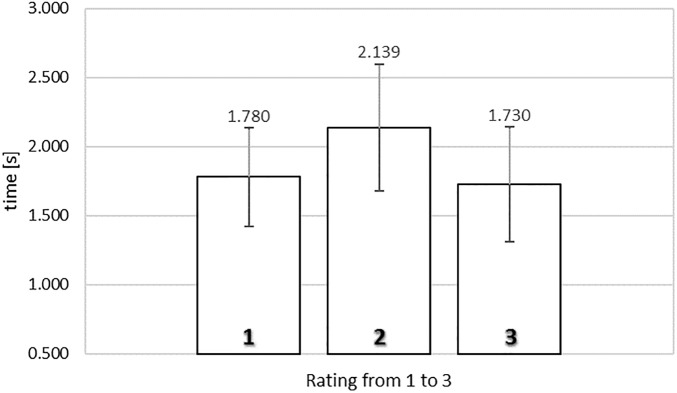
Average actual viewing time of naturalistic food images rated from 1 (‘*not like at all*’) to 3 (‘*like very much*’) in the self-paced non-exclusive evaluation. Error bars reflect the 95% confidence interval around the mean.

Figure 3 shows the average actual viewing times of food images as a function of rating in the self-paced non-exclusive evaluation. A one-way repeated measures ANOVA was conducted with three levels of rating (from 1, ‘*not like at all*,’ to 3, ‘*like very much’*), using the average actual viewing times for each subject for each level of rating as dependent measure. Mauchly’s test of sphericity indicated that the assumption of sphericity had not been violated, χ^2^(2) = 4.947, *p* = 0.084.

We obtained a significant relationship between rating and actual viewing time, *F*(2,76) = 7.353, MSE = 0.264, $\eta_{p}^{2}$= 0.162, *p* < 0.005. To gain further insights in the observed relationship between rating and actual viewing time, we analyzed the polynomial contrasts. The same trend was observed as in the SPN exposure time results, the quadratic contrast being significant, *F*(1,38) = 19.184, MSE = 0.200, $\eta_{p}^{2}$= 0.335, *p* < 0.0001, but not the linear contrast, F < 1. Again, the data showed an inverted U-shape trend, where images that received a rating of 2 were associated with the longest gaze durations (2.139 s). In contrast, food images that received extreme ratings were associated with shorter viewing durations; here, 1.780 s for images rated 1 (‘*not like at all*’) and 1.729 s for images rated 3 (‘*like very much*’).

### Time-Controlled Non-exclusive Evaluation (TCN)

#### TCN Exposure Time

**FIGURE 4:**
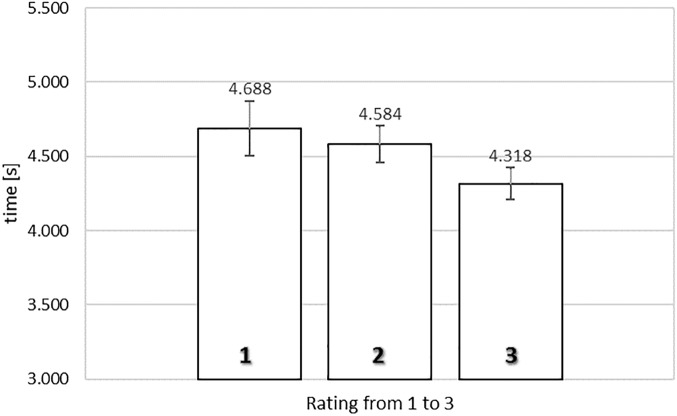
Average exposure time of food images rated from 1 (‘*not like at all*’) to 3 (‘*like very much*’) in the time-controlled non-exclusive evaluation. Error bars reflect the 95% confidence interval around the mean.

Figure 4 shows the average exposure times of food images as a function of rating in the time-controlled non-exclusive evaluation. A one-way repeated measures ANOVA was conducted, with three levels of rating (from 1, ‘*not like at all*,’ to 3, ‘*like very much*’), using the average exposure times for each subject for each level of rating as dependent measure. Mauchly’s Test of Sphericity indicated that the assumption of sphericity had not been violated, χ^2^(2) = 1.003, *p* = 0.606.

Our results indicated there was a significant relationship between rating and exposure time, *F*(2,78) = 5.500, MSE = 0.264, $\eta_{p}^{2}$ = 0.124, *p* < 0.01. To gain further insights in the observed relationship between rating and exposure time, we analyzed the polynomial contrasts. In the time-controlled non-exclusive evaluation task, the linear contrast was significant, *F*(1,39) = 9.995, MSE = 0.273, $\eta_{p}^{2}$ = 0.204, *p* = 0.003. However, the quadratic contrast was not significant, F < 1. The linear trend indicated that higher ratings were associated with shorter exposure durations (4.688 s for a rating of 1, 4.584 s for a rating of 2, and 4.318 s for a rating of 3).

#### TCN Actual Viewing Time

**FIGURE 5:**
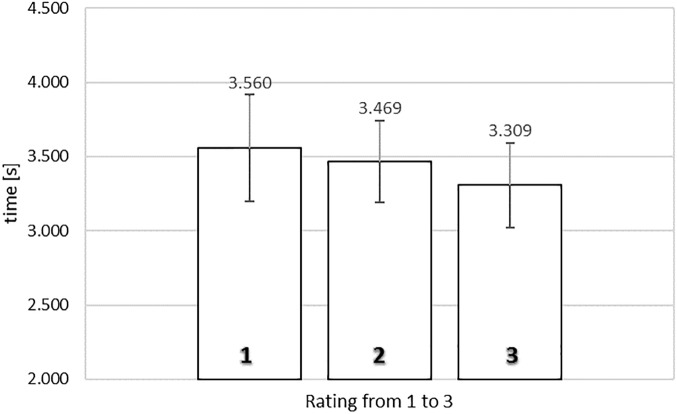
Average actual viewing time of naturalistic food images rated from 1 (‘*not like at all*’) to 3 (‘*like very much*’) in the time-controlled non-exclusive evaluation. Error bars reflect the 95% confidence interval around the mean.

Figure 5 shows the average actual viewing times of food images as a function of rating in the time-controlled non-exclusive evaluation. A one-way repeated measures ANOVA was performed with three levels of rating (from 1, ‘*not like at all*,’ to 3, ‘*like very much’*), using the average actual viewing times for each subject for each level of rating as dependent measure. Mauchly’s test of sphericity indicated that the assumption of sphericity had not been violated, χ^2^(2) = 2.663, *p* = 0.264.

Notably, there was no significant relationship between rating and actual viewing time, *F*(2,74) = 2.456, MSE = 0.251, *p* = 0.093. Therefore, our eye-tracking results indicate that the computer-controlled viewing conditions prevented a connection between gaze duration and non-exclusive evaluation.

### Self-Paced Exclusive Evaluation (SPE)

#### SPE Exposure Time

**FIGURE 6:**
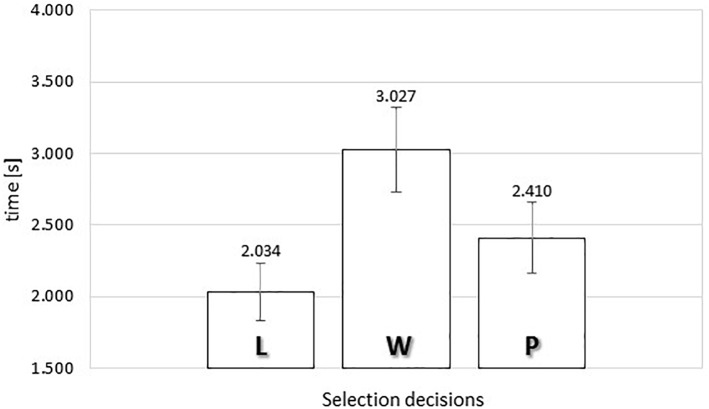
Average exposure time of food images as a function of selection decisions (“L” *leave it*, “W” *wish list*, or “P” *put it*) in the self-paced exclusive evaluation. Error bars reflect the 95% confidence interval around the mean.

Figure 6 presents the average exposure times of food images as a function of response category in the self-paced exclusive evaluation. A one-way repeated measures ANOVA was conducted, comparing the average exposure times for each subject for the response categories ‘*put it*,’ ‘*wish list*,’ and ‘*leave it*.’ Mauchly’s test of sphericity indicated that the assumption of sphericity had been violated, χ^2^(2) = 8.635, *p* = 0.013, implying that the conventional F-ratio for the one-way ANOVA could be too liberal. Therefore, we adopted the Huynh–Feldt correction, giving a more conservative test, *F*(1.727,67.357) = 11.132, MSE = 1.113, $\eta_{p}^{2}$ = 0.222, *p* < 0.0001. Thus, the test with the Huynh–Feldt correction confirmed that there was a significant relationship between the response categories and the exposure durations.

Following up with the polynomial contrasts, we noted that both the linear and quadratic contrasts were statistically significant, *F*(1,39) = 4.972, MSE = 0.570, $\eta_{p}^{2}$ = 0.113, *p* < 0.05 (linear contrast), and *F*(1,39) = 13.727, MSE = 1.353, $\eta_{p}^{2}$ = 0.260, *p* < 0.005 (quadratic contrast). Our data suggested an inverted U-shape trend, such that the non-committal ‘*wish list*’ response category was associated with the longest exposure durations (M_WISH_ = 3.057 s).

#### SPE Actual Viewing Time

**FIGURE 7:**
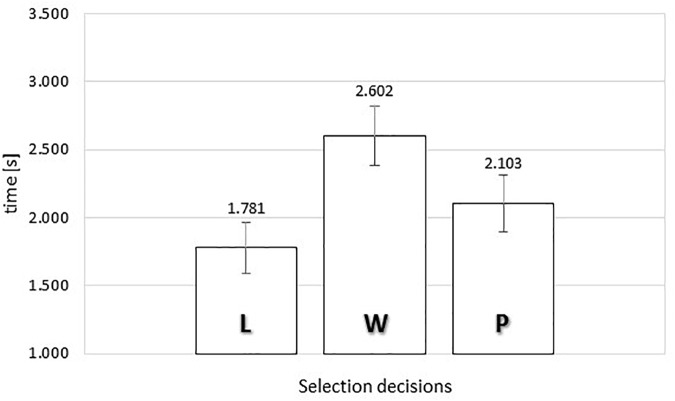
Average actual viewing time of food images as a function of selection decisions (“L” *leave it*, “W” *wish list*, or “P” *put it*) in the self-paced exclusive evaluation. Error bars reflect the 95% confidence interval around the mean.

Figure 7 presents the average actual viewing times of naturalistic food images as a function of response category. A one-way repeated measures ANOVA was conducted, comparing the average gaze durations for each subject for all three types of responses. Mauchly’s Test of Sphericity indicated that the assumption of sphericity had not been violated, χ^2^(2) = 1.520, *p* = 0.468.

The data indicated a significant relationship between response category and actual viewing time, *F*(2,74) = 11.098, MSE = 0.625, $\eta_{p}^{2}$ = 0.231, *p* < 0.0001. To gain further insights in the observed relationship between rating and actual viewing time, we analyzed the polynomial contrasts. The linear contrast was not significant, *F*(1,37) = 3.753, MSE = 0.523, *p* = 0.060. However, the quadratic contrast was statistically significant, *F*(1,37) = 16.385, MSE = 0.727, $\eta_{p}^{2}$ = 0.307, *p* < 0.0001. Once more, the data showed an inverted U-shape trend, where images that received the non-committal evaluation were associated with the longest gaze durations (2.628 s). In contrast, food images that received definite positive or negative evaluations were associated with shorter viewing durations (1.781 s for rejections and 2.103 s for inclusions).

### Time-Controlled Exclusive Evaluation (TCE)

#### TCE Exposure Time

**FIGURE 8:**
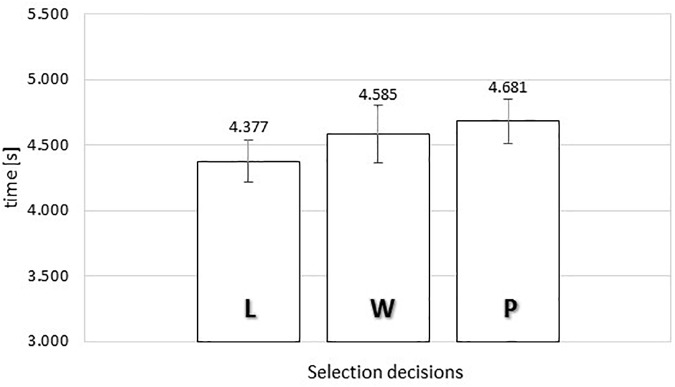
Average exposure time of food images as a function of selection decisions (“L” *leave it*, “W” *wish list*, or “P” *put it*) in the time-controlled exclusive evaluation. Error bars reflect the 95% confidence interval around the mean.

Figure 8 presents the average exposure times of food images as a function of response category in the time-controlled exclusive evaluation. We conducted a one-way repeated measures ANOVA, comparing the average exposure times for each subject for ‘put it,’ ‘wish list,’ and ‘leave it’ responses. Mauchly’s Test of Sphericity indicated that the assumption of sphericity had not been violated, χ^2^(2) = 5.440, *p* = 0.066.

The ANOVA produced no significant relationship between response category and exposure time, *F*(2,76) = 1.858, MSE = 0.507, *p* = 0.163.

#### TCE Actual Viewing Time

**FIGURE 9:**
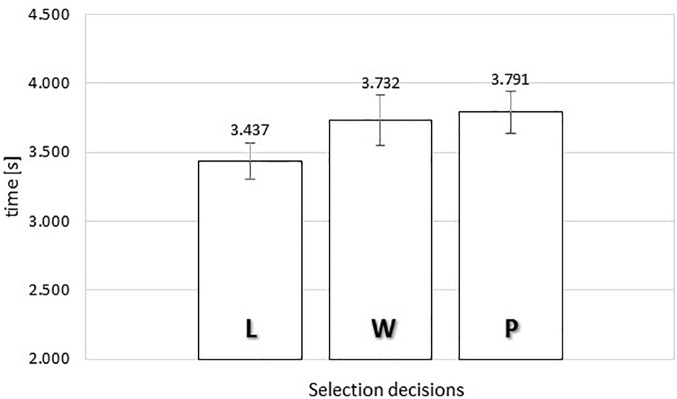
Average actual viewing time of food images as a function of selection decisions (“L” *leave it*, “W” *wish list*, or “P” *put it*) in the time-controlled exclusive evaluation. Error bars reflect the 95% confidence interval around the mean.

Figure 9 presents the average actual viewing times of food images as a function of response category in the time-controlled exclusive evaluation. One-way repeated measures ANOVA was performed, comparing the average exposure times for each subject for all three response categories (‘*leave it*,’ ‘*wish list*,’ and ‘*put it*’). Mauchly’s test of sphericity indicated that the assumption of sphericity had not been violated, χ^2^(2) = 4.855, *p* = 0.088.

Our results indicated that in the time-controlled exclusive evaluation there was a significant relationship between response category and gaze duration, *F*(2,72) = 3.535, MSE = 0.377, $\eta_{p}^{2}$ = 0.089, *p* < 0.05. To gain further insights in the observed relationship between evaluation and actual viewing time, we analyzed the polynomial contrasts. The linear contrast was significant, *F*(1,36) = 9.226, MSE = 0.250, $\eta_{p}^{2}$ = 0.204, *p* < 0.005, but not the quadratic contrast, F < 1. The linear trend indicated that longer gaze durations were associated with higher evaluations, with more than 300 ms extra gaze time for inclusions (M_PUT_ = 3.791 s) as compared to rejections (M_LEAV E_ = 3.437 s).

## Discussion

The present study set out to examine the relationship between viewing and preference formation with regard to single food images, using a similar 2 × 2 design as in our previous work (Wolf et al., 2018). Again, we included evaluation tasks either with or without self-determination of viewing time (i.e., self-paced or time-controlled), and either with or without limitations on the number of positive evaluations (i.e., exclusive or non-exclusive). In our earlier study we had obtained evidence against the dominant proposal that looking leads to liking during evaluative decision-making (e.g., Shimojo et al., 2003; Krajbich et al., 2010). However, our earlier study also raised a new set of questions about the divergent pattern of results. Particularly, we had found that looking was associated with liking in the exclusive evaluation task, whereas in the non-exclusive evaluation task, there was an inversed U-shape relationship between looking and liking. This divergence could be due to either the response set size (two versus five options) or to the existence of opportunity costs (limited or unlimited number of positive evaluations). To disambiguate these possibilities, we conducted the current study with a fixed set size of three options.

The present data pattern confirms once again that there is no general facilitative link between viewing time and liking during the evaluative processing of single food images. Under conditions of self-determination, we obtained inversed U-shape relationships between looking and liking in both the exclusive and non-exclusive evaluation tasks; the pattern of results was highly robust, measured both in terms of exposure durations and actual viewing times. Under time-controlled viewing conditions, when the subjects could not determine the duration of stimulus presentation, the relationships between viewing and liking were markedly less robust, with null effects for actual viewing times in the time-controlled non-exclusive evaluation task and for exposure durations in the time-controlled exclusive evaluation task.

### Self-Determination of Viewing Time

As in our previous study (Wolf et al., 2018), the ability to determine the duration of stimulus presentation had a critical impact on the current pattern of results. Here, we found inversed U-shaped relationships between viewing and evaluation only in the self-paced evaluation tasks. To the extent that there were still significant associations between looking and liking in the time-controlled conditions, they were reflected as linear relationships. Thus, the patterns were significantly altered by the self-determination of viewing time. Taken together with our previous study, the findings indicate that the voluntary control over the presentation of stimulus duration is a critical factor that *enables* a robust relationship between viewing and evaluative processing. In all self-paced evaluations across the two studies, we always obtained the same highly significant patterns of results for exposure durations and gaze durations. In contrast, the time-controlled evaluations are less robust. In our previous study, we found no significant effects whenever the presentation of the images was set automatically to a pseudo-random duration; in the current study, the time-controlled evaluations produced inconsistent effects, for exposure duration but not for gaze duration in the non-exclusive evaluation, and vice versa in the exclusive evaluation.

### Slow Viewing for the Middle Category

In the self-paced conditions, we obtained inverted U-shaped trends for both the exclusive and non-exclusive evaluations. Thus, given a fixed response set size of three options, there was a similar relationship between viewing and evaluation for both types of evaluation. In our previous study (Wolf et al., 2018), we found an inverted U-shaped trend in the non-exclusive evaluation, when there were five response options, whereas it was impossible to observe such a relationship in the exclusive evaluation, with only two response options (exclude or include). In the present study, with three options for both exclusive and non-exclusive evaluations, it turned out that categorically positive or negative evaluations were always associated with shorter viewing times than the middle category (‘2’ in the non-exclusive evaluation task; ‘*wish list*’ in the exclusive evaluation task).

Here, we suggest that the observation of slow viewing for the middle category may be analogous to phenomena such as increased selective attention and sustained stimulus processing under uncertainty (Dieterich et al., 2016), or extended information gathering with higher decision thresholds (Hauser et al., 2017). By this interpretation, the longer viewing durations for items that receive a non-committal evaluation are reflective of doubt or indecisiveness, with subjects unable to reach a quick positive or negative evaluation. More specifically, we suggest that the speeded polarized evaluations are possible because subjects work on the basis of internal criteria for what constitutes an appetitive food image versus what constitutes an aversive food image. For instance, one might envisage a value-based decision mechanism with separate thresholds requiring a specific level of positive affect for a positive decision and a specific level of negative affect for a negative decision. Subjects would view the images until a decision can be triggered. In cases when no strong positive or negative affect emerges, no threshold can be reached, effectively equivalent to a state of uncertainty or indecision, with subjects continuing to scrutinize the images in an attempt to discern visual features that are relevant for affective processing. It might require a metacognitive decision mechanism to resolve the indecision, by concluding that neither a positive nor a negative evaluation can be reached, so that the non-committal response option can be activated (‘2’ in the non-exclusive evaluation task; ‘*wish list*’ in the exclusive evaluation task).

With respect to the response options, it should be noted as a limitation of the current study that, in order to match the set size in the non-exclusive and exclusive evaluations, the liking rating was reduced to just three options. Normal liking ratings are made on continuous scales, or Likert scales with seven or nine options. Using three-point scales might have elicited dichotomous thinking. Also, the exclusive evaluation, by which subjects could only choose 15 items from a list presented serially, poses a challenge as the decision-maker has no knowledge of the likely value of subsequent items so the threshold for exclusion or inclusion is likely to change across the course of the experimental session.

A further limitation of the present study is that any individual differences in food preferences may derive from different cognitive styles with respect to processing information about food (e.g., dieters may focus on their dietary restrictions as they evaluate food items). Our research question is concerned with viewing time as a more general index of the extent of information processing, without making any assumptions on the cognitive content. However, the notion that the relationship between viewing time and liking should hold similarly for any subject, regardless of their cognitive style with respect to food, must be recognized as a presumption, to be put to the test in future research.

### A Potential Role for Opportunity Costs

The major finding of the present study, taken together with our previous study, unambiguously pointed to the factor of response set size as a critical determinant for the relationship between viewing and preference formation in the self-paced exclusive versus non-exclusive evaluation tasks. However, we cannot rule out a potential additional role for opportunity costs. As argued in the Introduction, opportunity costs were hypothesized to elicit a type of cumulative processing for decision-making that inherently links positive decisions with longer viewing times, particularly when positive decisions come at the expense of the exclusion of other items. Interestingly, our present findings do not convincingly rebut this hypothesis.

Separately from any inversed-U shaped relationships (as indicated by significant quadratic contrasts), we obtained linear relationships suggestive of a connection between longer viewing and increased evaluation for the exposure durations in the self-paced exclusive evaluation and for the gaze durations in the time-controlled exclusive evaluation. The results were inconsistent in the sense that the significance tests for the gaze durations and exposure durations did not match perfectly. However, visual inspection of the data suggests that the inconsistency in significance testing was due to the limits of statistical power in our study. The differences between the ‘*leave it*’ versus ‘*put it*’ responses were more than 300 ms in all cases; always slower for the positive evaluations (Figure 6–9). Conversely, in the absence of opportunity costs, the differences between the ‘1’ and ‘3’ responses were smaller, and always faster for positive evaluations (Figure 2–5).

One possibility is that the opportunity costs in the exclusive evaluation tasks were not large enough to elicit robust effects, given the present statistical power. It could be that the restriction of 15 out of 80 items, with the added option of placing items on a wish list, did not make the opportunity costs salient enough for our subjects. Therefore, in future work we aim to investigate the role of opportunity costs in the relationship between viewing and preference formation more directly, by varying the level of restriction on positive evaluations.

## Conclusion

The present findings provided further evidence that the purported facilitation from looking to increased liking does not hold true in general. Instead, we propose that self-determination of viewing time enables the connection between viewing and evaluative processing. This connection is complex, with relatively short viewing times for highly positive or negative evaluations, but slow viewing times for neutral or non-committal evaluations, suggesting that uncertainty or indecision can prolong the efforts of visual processing.

## Data Availability

All datasets generated for this study are included in the manuscript and the Supplementary Files (see Supplementary Data Sheet S1).

## Ethics Statement

This study was approved by the Human Ethics Committee of the Faculty of Arts and Science, Kyushu University (Issue No. 201801). Informed consent was obtained in writing from each subject.

## Author Contributions

AW conducted the data collection for the study, analyzed the behavior and eye-tracking data, and prepared all figures. AW and KO programmed the experiments. AW and JL wrote the manuscript. All authors reviewed and approved the manuscript and contributed to the design of the study.

## Conflict of Interest Statement

The authors declare that the research was conducted in the absence of any commercial or financial relationships that could be construed as a potential conflict of interest.

## Supplementary Material

DATA SHEET S1 Complete data set for the present study, organized in four Data Sheets: SPN_123 (with exposure durations, gaze durations, and number of trials per response type in the self-paced non-exclusive evaluation); TCN_123 (with exposure durations, gaze durations, and number of trials per response type in the time-controlled non-exclusive evaluation); SPE_LWP (with exposure durations, gaze durations, and number of trials per response type in the self-paced exclusive evaluation); and TCE_LWP (with exposure durations, gaze durations, and number of trials per response type in the time-controlled exclusive evaluation).
